# Odyssey of human dental pulp stem cells and their remarkable ability to survive in extremely adverse conditions

**DOI:** 10.3389/fphys.2015.00099

**Published:** 2015-03-26

**Authors:** Thimios A. Mitsiadis, Anna Woloszyk

**Affiliations:** Orofacial Development and Regeneration, Faculty of Medicine, Institute of Oral Biology, Center for Dentistry, University of ZurichZurich, Switzerland

**Keywords:** tooth, dental pulp stem cells, tissue regeneration, stem cells, stem cell niches, quiescent stem cells, hypoxia

Adult tissues contain stem cells, which proliferate to compensate for tissue loss throughout the life of the organism (Li and Clevers, 2010; Jiménez-Rojo et al., 2012). Therefore, adult stem cells are important for tissue repair and regeneration after injury. In seriously injured or carious teeth, stem cells residing in the dental pulp are responsible for the repair and regeneration of the damaged dental tissues (Catón et al., 2011; Mitsiadis et al., 2011). Dental pulp stem cells (DPSCs) can be isolated after labeling of the pulp cells with fluorescent mesenchymal stem cell (MSC) markers such as STRO-1, CD146, and CD44, followed by fluorescence-activated cell sorting (FACS) (Mitsiadis et al., 2012). Additional identification procedures include morphological criteria, adherence properties, proliferation, and differentiation potential, as well as tissue repair abilities of the sorted stem cell populations. The potential of DPSCs for the regeneration of bone tissue in patients has been demonstrated with a successful trial assay that has been realized in Napoli (Italy) several years ago (Catón et al., 2011; Mitsiadis et al., 2012).

We have established a close scientific collaboration with the Napoli stem cell team headed by Prof. Gianpaolo Papaccio for the exchange of knowledge and materials. Several months ago, we have requested for a bunch of freshly isolated DPSCs in order to complete a number of experiments that we are currently realizing in our laboratories in Zurich (Switzerland). DPSCs were shipped with an express delivery company on the 26/11/2014 from Napoli with destination to Zurich. The shipped DPSCs were grown as a monolayer in a 25 cm^2^ culture flask containing the standard MSC culture medium. The flask that was sealed with Parafilm and left in ambient temperature should normally arrive to the destination the next day, but for unknown reasons the journey of DPSCs was longer and more exciting than expected. The DPSCs have followed an astonishing itinerary from Italy, to France (Paris), Great Britain, South Africa (Johannesburg), back to France, thereafter to Spain, France again. From France the cells finally arrived to Zurich Switzerland through Basel on the 4/12/2014. This itinerary can be considered as an Odyssey of DPSCs, which had to develop sophisticated mechanisms for their survival under these extremely adverse conditions.

Sometimes discoveries are arising by chance, following a mistake or having an unexpected behavior. It was expected that the flask, upon its late arrival, would contain only dead cells. However, surprisingly, DPSCs (or a big part of them) were still alive after their journey (Figure 1A). DPSCs were extremely proliferative and became confluent within 3 days, after changing the medium (Figures 1B,C). How this could be explained? Recent findings indicate that quiescent (reserve) and proliferating (active) stem cell pools may coexist in separate but adjacent compartments of many tissues (Li and Clevers, 2010). It has been proposed that stem cells can adopt a reversible quiescence state characterized by reduced metabolic activity in conditions such as lack of oxygen and/or nutrients (Cheung and Rando, 2013). However, the mechanisms that allow quiescent stem cells to survive metabolic or environmental stress, to preserve their cellular and genomic integrity and to assure long-term survival are not yet elucidated. It has been suggested that quiescence is an actively maintained state regulated by intrinsic mechanisms to sustain metabolic function during persistent environmental stress, and thus ensuring stem cell survival (Cheung and Rando, 2013). Viable stem cells were found in post-mortem tissues (Latil et al., 2012). These cells were able to maintain their functional properties after prolonged storage in anoxia *in vitro* and after transplantation (Latil et al., 2012). Similarly, it has been shown that several stem cell populations reside in poorly oxygenated niches (Simsek et al., 2010). Quiescent stem cells have the ability to sense environmental changes and respond by re-entering the cell cycle for proliferation (Cheung and Rando, 2013). Severe hypoxia could be critical for maintaining the viability of DPSCs. It is possible that mechanisms compatible with the low metabolic state of quiescence have allowed rapid responses for DMSCs re-activation.

**Figure 1:**
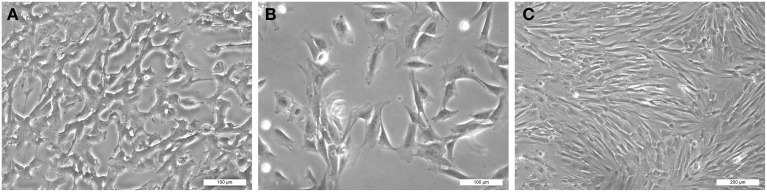
**Visualization of human dental pulp stem cells (hDPSCs): (A) upon arrival to Zurich (first passage), (B) after 6 h of culture (second passage), and (C) after 3 days of culture (second passage)**. Scale bars indicate the magnifications.

This Odyssey revealed that DPSCs are able to survive for prolonged periods of time in conditions of extreme stress.

## Conflict of interest statement

The authors declare that the research was conducted in the absence of any commercial or financial relationships that could be construed as a potential conflict of interest.
